# DTI measures track and predict motor function outcomes in stroke rehabilitation utilizing BCI technology

**DOI:** 10.3389/fnhum.2015.00195

**Published:** 2015-04-27

**Authors:** Jie Song, Veena A. Nair, Brittany M. Young, Leo M. Walton, Zack Nigogosyan, Alexander Remsik, Mitchell E. Tyler, Dorothy Farrar-Edwards, Kristin E. Caldera, Justin A. Sattin, Justin C. Williams, Vivek Prabhakaran

**Affiliations:** ^1^Department of Biomedical Engineering, University of Wisconsin–Madison, Madison, WIUSA; ^2^Department of Radiology, University of Wisconsin–Madison, Madison, WIUSA; ^3^Neuroscience Training Program, University of Wisconsin–Madison, Madison, WIUSA; ^4^Medical Scientist Training Program, University of Wisconsin–Madison, Madison, WIUSA; ^5^Department of Orthopedics and Rehabilitation, University of Wisconsin–Madison, Madison, WIUSA; ^6^Departments of Kinesiology, University of Wisconsin–Madison, Madison, WIUSA; ^7^Departments of Medicine, University of Wisconsin–Madison, Madison, WIUSA; ^8^Department of Neurology, University of Wisconsin–Madison, Madison, WIUSA; ^9^Department of Neurosurgery, University of Wisconsin–Madison, Madison, WIUSA; ^10^Department of Psychiatry, University of Wisconsin–Madison, Madison, WIUSA; ^11^Department of Psychology, University of Wisconsin–Madison, Madison, WIUSA

**Keywords:** DTI, fractional anisotropy, axial diffusivity, radial diffusivity, mean diffusivity, motor recovery, stroke rehabilitation, brain-computer interface

## Abstract

Tracking and predicting motor outcomes is important in determining effective stroke rehabilitation strategies. Diffusion tensor imaging (DTI) allows for evaluation of the underlying structural integrity of brain white matter tracts and may serve as a potential biomarker for tracking and predicting motor recovery. In this study, we examined the longitudinal relationship between DTI measures of the posterior limb of the internal capsule (PLIC) and upper-limb motor outcomes in 13 stroke patients (median 20-month post-stroke) who completed up to 15 sessions of intervention using brain–computer interface (BCI) technology. Patients’ upper-limb motor outcomes and PLIC DTI measures including fractional anisotropy (FA), axial diffusivity (AD), radial diffusivity (RD), and mean diffusivity (MD) were assessed longitudinally at four time points: pre-, mid-, immediately post- and 1-month-post intervention. DTI measures and ratios of each DTI measure comparing the ipsilesional and contralesional PLIC were correlated with patients’ motor outcomes to examine the relationship between structural integrity of the PLIC and patients’ motor recovery. We found that lower diffusivity and higher FA values of the ipsilesional PLIC were significantly correlated with better upper-limb motor function. Baseline DTI ratios were significantly correlated with motor outcomes measured immediately post and 1-month-post BCI interventions. A few patients achieved improvements in motor recovery meeting the minimum clinically important difference (MCID). These findings suggest that upper-limb motor recovery in stroke patients receiving BCI interventions relates to the microstructural status of the PLIC. Lower diffusivity and higher FA measures of the ipsilesional PLIC contribute toward better motor recovery in the stroke-affected upper-limb. DTI-derived measures may be a clinically useful biomarker in tracking and predicting motor recovery in stroke patients receiving BCI interventions.

## Introduction

One of the most common deficits following stroke is upper-limb motor impairment, which can have a significant impact on disability and health (Coupar et al., 2012). Rehabilitation is often scheduled for patients to minimize disability and to improve the quality of patients’ daily living activities. Estimating patient’s potential for motor function recovery is critical in making decisions on the type, duration and goals of rehabilitation (Stinear, 2010). Previous studies have reported that stroke location rather than the volume of an infarct was more important for predicting functional recovery (Pineiro et al., 2000; Crafton et al., 2003). Furthermore, the posterior limb of the internal capsule (PLIC) was significantly associated with poor recovery of isolated upper-limb movements (Shelton and Reding, 2001). Given the significance of PLIC involved in motor impairment and recovery, one aim of this study is to systematically assess the structural integrity of the PLIC using diffusion tensor imaging (DTI). DTI is a non-invasive brain imaging technique that allows for quantitative evaluation of the structural integrity of white matter tracts after a stroke (Werring et al., 2000; Stinear et al., 2007; Yu et al., 2009). A recent study shows evidence that DTI measures may be used as potential biomarkers for predicting stroke recovery in stroke patients receiving transcranial direct current stimulation (Lindenberg et al., 2012). BCI facilitated intervention is a novel neurorehabilitation therapy that has drawn increasing attention in recent years. BCI devices provide real-time feedback to assist users to learn modulation of brain activity. This type of modulation and further enhancement with motor training may promote brain plasticity changes and eventually boost the recovery when patients have reached a functional plateau with more traditional therapies (Fallani et al., 2013; Varkuti et al., 2013). To date, few studies have examined the white matter structural integrity in stroke patients receiving BCI intervention.

We have recently proposed that FA is a valuable measure for examining the microstructural integrity of the PLIC and a promising biomarker in tracking and predicting motor functional recovery in stroke patients receiving BCI interventions (Song et al., 2014). In this study, we systematically and longitudinally examined the structural integrity of the PLIC in 13 stroke patients who completed up to 15 sessions of BCI intervention facilitated by functional electrical muscle stimulation (FES). This analysis included measures of fractional anisotropy (FA), axial diffusivity (AD), radial diffusivity (RD), and mean diffusivity (MD) and ratio of each of these measures between the ipsilesional and contralesional PLIC. We further evaluated the predictive value of these DTI measures on upper-limb motor impairment and functional recovery in stroke patients receiving BCI interventions to determine whether they are clinically meaningful predictors of motor recovery.

## Materials and Methods

### Patients

Thirteen patients with stroke were included into this study. This study was approved by the University of Wisconsin-Madison’s Institutional Review Board. All patients provided written informed consent. Standard clinical MRI was utilized to assess damage to PLIC by a neuroradiologist (VP). Ten of the 13 patients showed damage to the PLIC due to stroke. Patients CI003 with a minor left frontal lobe infarct, CI010 with right occipital stroke and CT003 with a right pontine infarct did not show damage to the PLIC. Patient CT004 with a left middle cerebral artery (MCA) territory infarct showed minimal damage to the PLIC. A summary of patient characteristics is given in **Table 1**.

**Table 1:** Patient characteristics.

Patient ID	Age/years	Gender	Months since stroke	Baseline NIHSS	Baseline NIHSS-motor arm	Lesion location
CI001	52	M	15	8	4	Left MCA
CI002	62	F	16	8	4	Left precentral gyrus
CI003	68	M	3	0	0	Left frontal lobe
CI004	66	M	23	6	1	Left MCA
CI005	73	F	2	0	0	Left MCA
CI007	59	M	28	2	0	Left temporal lobe
CI008	45	F	99	6	2	Right frontoparietal infarct
CI009	71	F	26	6	2	Right temporal-frontal-parietal lobe
CI010	80	M	20	2	0	Right occipital lobe
CT001	75	F	23	7	3	Right putamen
CT002	55	M	17	0	0	Left basal ganglia
CT003	49	M	6	3	1	Right pons
CT010	50	M	26	4	3	Right MCA
Mean ± SD	61.9 ± 11.21	5 F/8 M	23.4 ± 24.33	4.0 ± 3.03	1.5 ± 1.56	7 L/ 6 R

*MCA, middle cerebral artery.*

### Study Design

Diffusion tensor imaging data and motor outcome assessments were acquired at four time points: before the start of intervention (i.e., immediately pre-intervention), at the midpoint of intervention (i.e., mid-intervention), upon completion of intervention phase (i.e., immediately post-intervention), and 1 month following the last session of BCI intervention (i.e., 1-month-post-intervention). A detailed description of the study design and the procedure of BCI intervention is elaborated in references (Song et al., 2014; Young et al., 2014a,b). Each patient was administered up to fifteen 2-h sessions of interventional BCI therapy (14.08 ± 1.71 sessions). These sessions took place over a period of up to 6 weeks with two to three intervention sessions per week.

Patient inclusion criteria include: (1) ages 18 years and above; (2) no known neurologic, psychiatric or developmental disability; (3) persistent upper-extremity motor impairment resulting from ischemic or hemorrhagic stroke. Exclusion criteria include: (1) contraindications for MRI; (2) allergy to electrode gel, surgical tape and metals that would be used in BCI intervention; (3) under treatment for infectious disease or having apparent oral lesions or inflammation.

### Motor Outcome Measures

Each patient was assessed for clinical stroke severity in addition to neurological examination at four time points throughout the BCI intervention. The overall neurological deficit and severity of motor paresis was evaluated using the National Institute of Health Stroke Scale (NIHSS; Brott et al., 1989). Upper-limb functional performance was assessed by the Action Research Arm Test (ARAT). The ARAT is a standardized performance measure of upper-limb function assessing grasp, grip, pinch and gross arm movement. The ARAT scores range from 0 to 57. Besides an objective evaluation of motor outcomes using ARAT, we included a subjective measure of the Stroke Impact Scale (SIS) hand function subscale (SIS-hand function). SIS-hand function was used to assess self-reported satisfaction with hand use. Raw scores of SIS-hand function were transformed (Stewart and Ware, 1992) with a transformed scale = 100 × [(actual raw score – lowest possible raw score)/possible raw score]. The transformed SIS-hand function scores range from 0 to 100. All clinical assessments of the stroke-affected limb for each patient at each time point are given in **Table 2**.

**Table 2:** Clinical motor outcome assessments of the stroke-affected limb.

Subject ID	Time points	ARAT	SIS Hand function
CI001	Pre-therapy	0	0
	Mid-therapy	3	0
	Immediately post-therapy	0	0
	One-month-post-therapy	0	0
CI002	Pre-therapy	0	0
	Mid-therapy	0	0
	Immediately post-therapy	0	0
	One-month-post-therapy	0	0
CI003	Pre-therapy	57	40
	Mid-therapy	57	55
	Immediately post-therapy	57	70
	One-month-post-therapy	57	75
CI004	Pre-therapy	3	0
	Mid-therapy	0	0
	Immediately post-therapy	0	0
	One-month-post-therapy	0	0
CI005	Pre-therapy	56	50
	Mid-therapy	46	70
	Immediately post-therapy	54	50
	One-month-post-therapy	57	57.5
CI007	Pre-therapy	57	55
	Mid-therapy	57	100
	Immediately post-therapy	57	70
	One-month-post-therapy	57	65
CI008	Pre-therapy	3	0
	Mid-therapy	5	0
	Immediately post-therapy	4	0
	One-month-post-therapy	5	0
CI009	Pre-therapy	3	0
	Mid-therapy	3	10
	Immediately post-therapy	0	5
	One-month-post-therapy	3	10
CI010	Pre-therapy	57	55
	Mid-therapy	54	60
	Immediately post-therapy	57	70
	One-month-post-therapy	57	70
CT001	Pre-therapy	0	5
	Mid-therapy	0	0
	Immediately post-therapy	0	0
	one-month-post-therapy	0	0
CT002	Mid-therapy	53	75
	Immediately post-therapy	57	75
	One-month-post-therapy	54	75
CT003	Pre-therapy	27	10
	Mid-therapy	28	30
	Immediately post-therapy	40	35
	One-month-post-therapy	43	45
CT010	Pre-therapy	3	20
	Mid-therapy	5	30
	Immediately post-therapy	4	15
	One-month-post-therapy	8	30

### DTI Data Acquisition and Processing

DTI data was acquired on a 3 T whole-body MRI scanner (GE DISCOVERY MR750, General Electric Medical Systems, Waukesha, WI, USA) with an 8-channel head coil. MR diffusion imaging parameters were: single-shot echo planar imaging (EPI), TR = 9000 ms, TE = 66.2 ms, single average (NEX = 1), field of view (FOV) = 256 × 256 mm^2^, voxel size = 1 × 1 × 2 mm^3^, 75 axial slices with no gap between slices, flip angle = 90°, 56 gradient encoded directions, *b* value = 1000 s/mm^2^.

**FIGURE 1:**
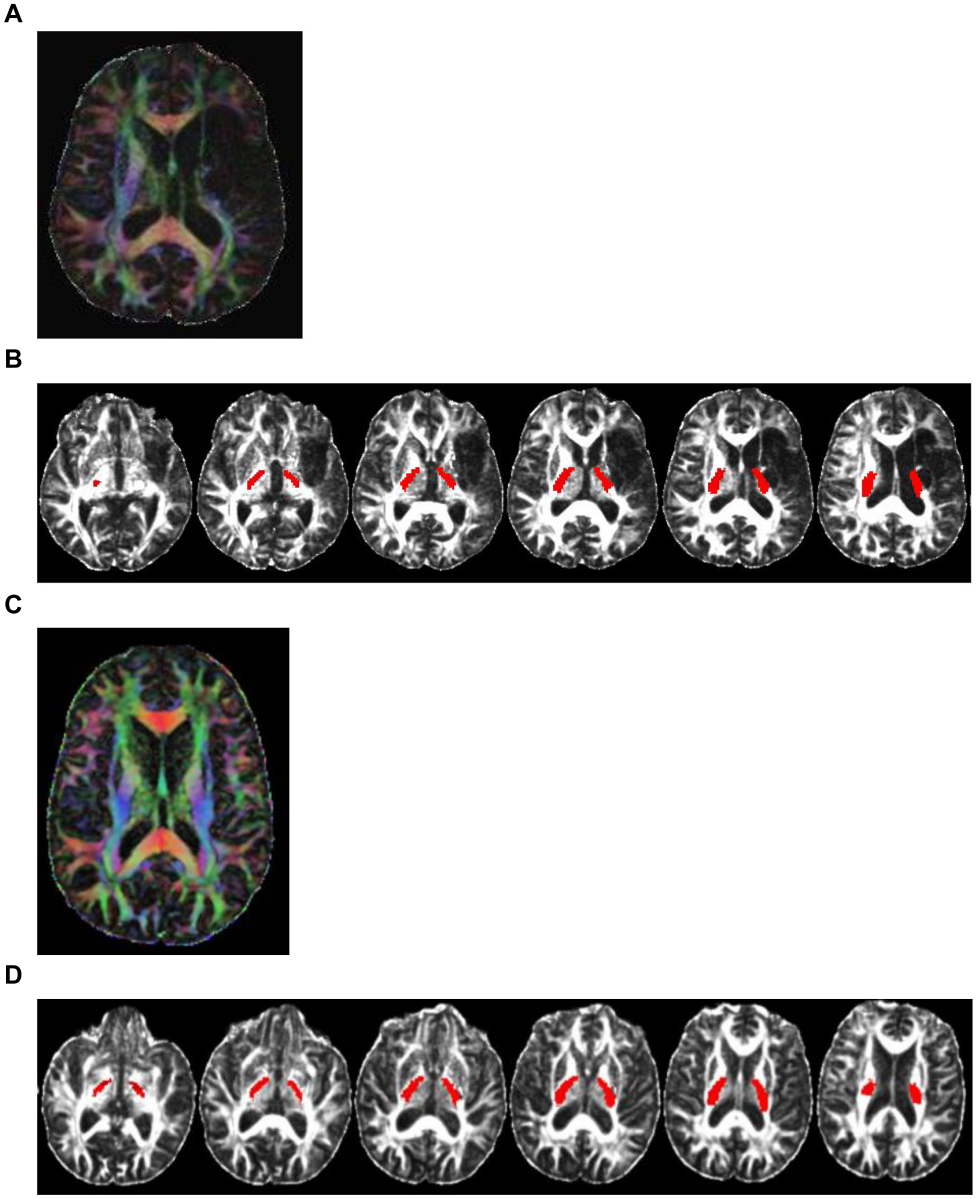
**Color-coded diffusion tensor imaging (DTI) images and the PLIC white-matter tract.** Directionally encoded color (DEC) map of DTI images and the PLIC white-matter tract. **(A)** A DTI image of a representative patient (CI001) with damage to PLIC. Color codes to give diffusion tensor directions: red represents tracts running left to right; green is anterior to posterior; blue is superior to inferior. **(B)** Axial view of the PLIC white-matter tract (in red) of patient CI001. **(C)** A DEC map of DTI image of a representative patient (CI003) with no damage to PLIC. Color also represents diffusion tensor directions. **(D)** Axial view of the PLIC white-matter tract (in red) of patient CI003.

DTI data was processed using FSL (v5.0; Smith et al., 2004) with the following steps: (1) correction for motion and eddy current distortion; (2) head-motion correction for gradient direction vectors; (3) image registration and brain mask extraction; (4) estimation of tensor diffusion and generating diffusivity measures; (5) registration of standard white-matter atlases (JHU-ICBM-FA-2mm.nii.gz and JHU-ICBM-labels-2mm.nii.gz) to each patient’s native space; step 5 provided automatically segmented white-matter tracts with corresponding labels for identifying the regions of interest such as PLIC (**Figure 1**); (6) estimation of diffusivity for each hemispheric PLIC; (7) calculation of ratios of DTI measures (rFA, rAD, rRD, rMD) between the ipsilesional and contralesional PLIC as rValue = Value_ipsi_/ Value_contra_ (Kusano et al., 2009; Koyama et al., 2012).

### Statistical Analyses

Statistical analysis was performed using RStudio (version 0.98.1062). The threshold for statistical significance was set to *p-value* ≤ 0.05. A one-sided Wilcoxon signed-rank test was used to compare each DTI measure between the ipsilesional and contralesional PLIC. A Kruskal–Wallis test which is a non-parametric equivalent of ANOVA was used to evaluate the time effect on the motor outcome performances during the course of intervention. Spearman’s rank correlation coefficients were computed to examine correlations between diffusivity and motor outcome measures. To take advantage of a longitudinal, repeated-measurement design of this study, we used a generalized estimating equation (GEE) for regression analysis (Ballinger, 2004). GEEs take into account the dependency of repeated measurements from the same patient in the regression analysis and thus allow for evaluation of the relationship between DTI and motor outcome measures longitudinally. A linear regression analysis was also computed to investigate whether baseline DTI measures predicted post-intervention motor outcomes.

## Results

### Patient Motor Outcome Measures

Motor outcome measures are summarized in **Table 2**. The ARAT scores varied from zero, indicating no ability to perform the task, to maximum of 57, indicating unimpaired performance. The SIS-hand function measure varied widely, with a value of zero indicating a patient reporting no ability to use the impaired hand, and higher positive values indicating decreasing levels of difficulty using the impaired hand in daily activities such as carrying heavy objects, turning a doorknob, opening a can or jar, tying a shoe lace and picking up a dime. Although group performance on the ARAT and SIS-hand function measures was not significantly different over the course of BCI intervention (Kruskal–Wallis test, *p*-value > 0.05), 7 of 13 patients reported improved hand function based on their SIS-hand function scores measured at 1-month-post intervention. Three of these seven patients (CI005, CI007, and CT003) also achieved improvements meeting the minimum clinically important difference (MCID) of SIS-hand function (cutoff value = 17.8; Lin et al., 2010) at mid-intervention. Similarly, 5 of 13 patients had improved ARAT measures at 1-month-post intervention compared to baseline measures. One patient (CT003) achieved improvements meeting the MCID of ARAT (cutoff value = 5.7) at 1-month-post intervention.

### Increased Diffusivity and Decreased FA Measures in Ipsilesional PLIC

**FIGURE 2:**
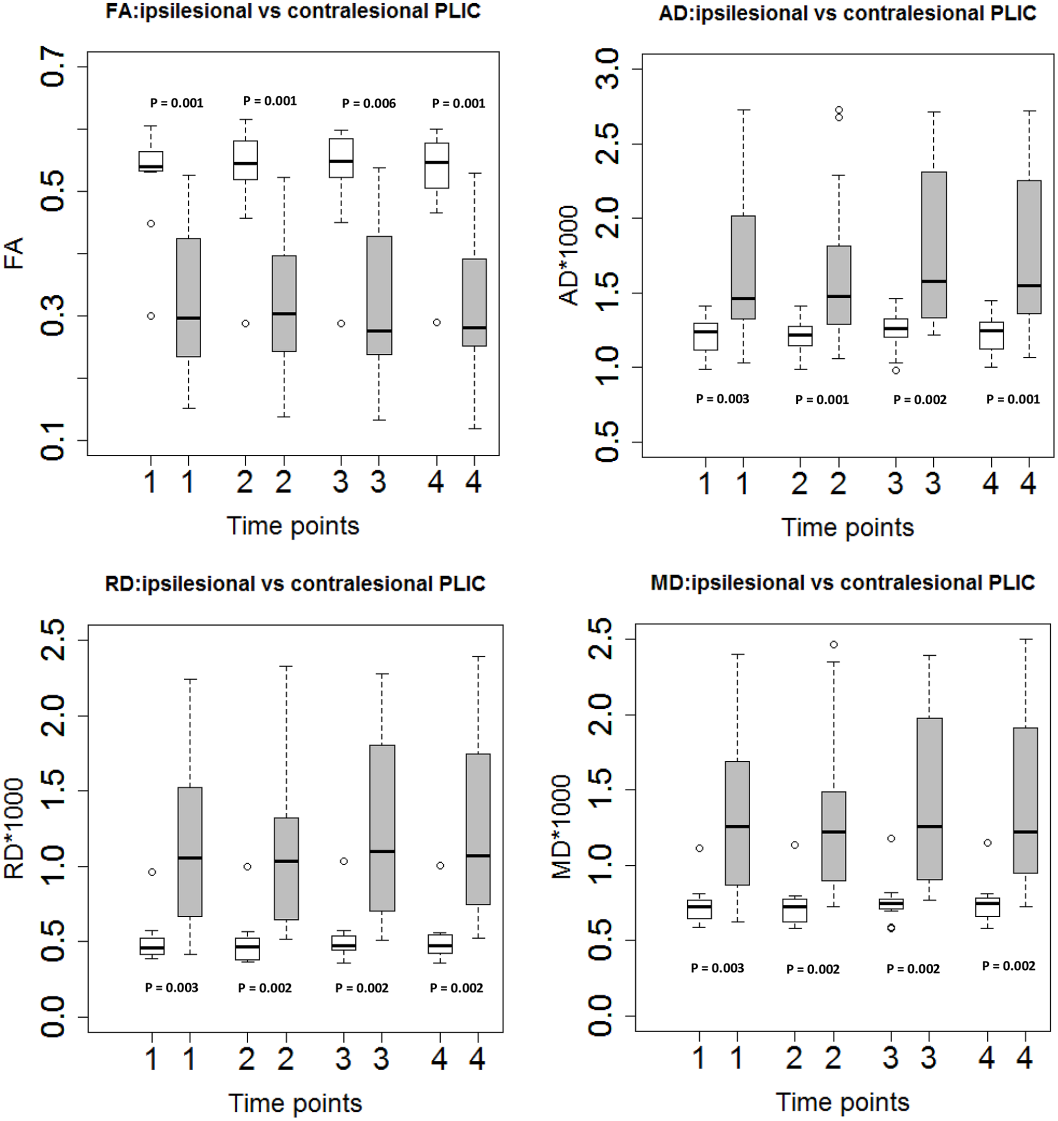
**Diffusion tensor imaging measures in ipsilesional and contralesional PLIC.** Boxplots showed significantly higher FA, lower diffusivity measures (AD, RD, and MD) in the ipsilesional PLIC (Wilcoxon signed-rank tests).Time of immediately pre-, mid-, immediately post- and 1-month-post intervention are indicated as time-point 1, 2, 3, and 4, respectively. White boxes represent the contralesional side and gray boxes represent the ipsilesional side of PLIC.

The mean values of AD, RD, and MD were higher on the ipsilesional PLIC than on the contralesional side, while FA was lower in the ipsilesional PLIC than in the controlesional PLIC (Wilcoxon signed-rank test, *p*-values < 0.05; **Figure 2**).

### Correlation of DTI Measures with Motor Functional Recovery

**FIGURE 3:**
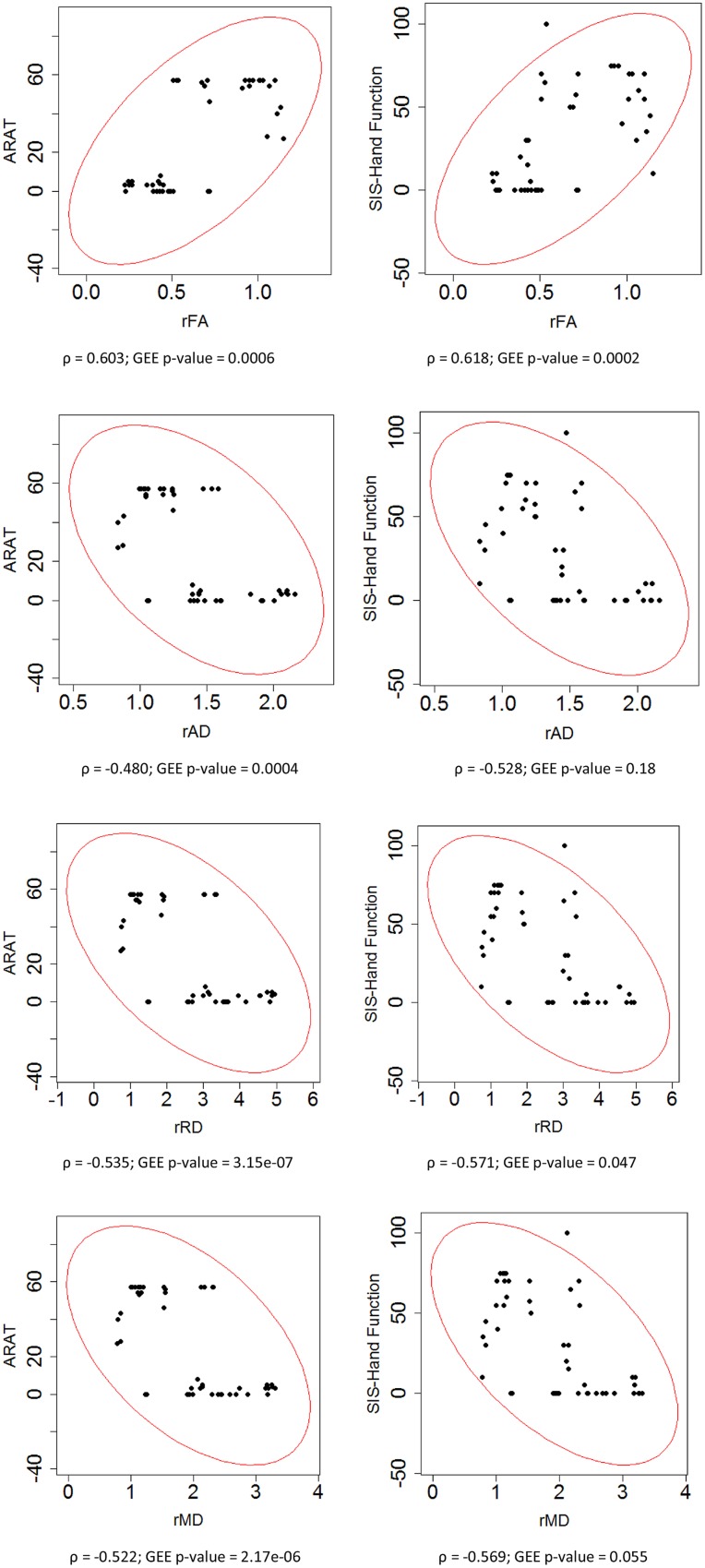
**Correlation of ratios of each DTI measure and motor outcomes across time.** Each ratio of DTI measures was correlated with motor outcome measures including ARAT and SIS hand function scores assessed in impaired hand. Spearman rank correlation coefficient (ρ) and the statistical significance of correlation (GEE *p*-value) are presented.

**FIGURE 4:**
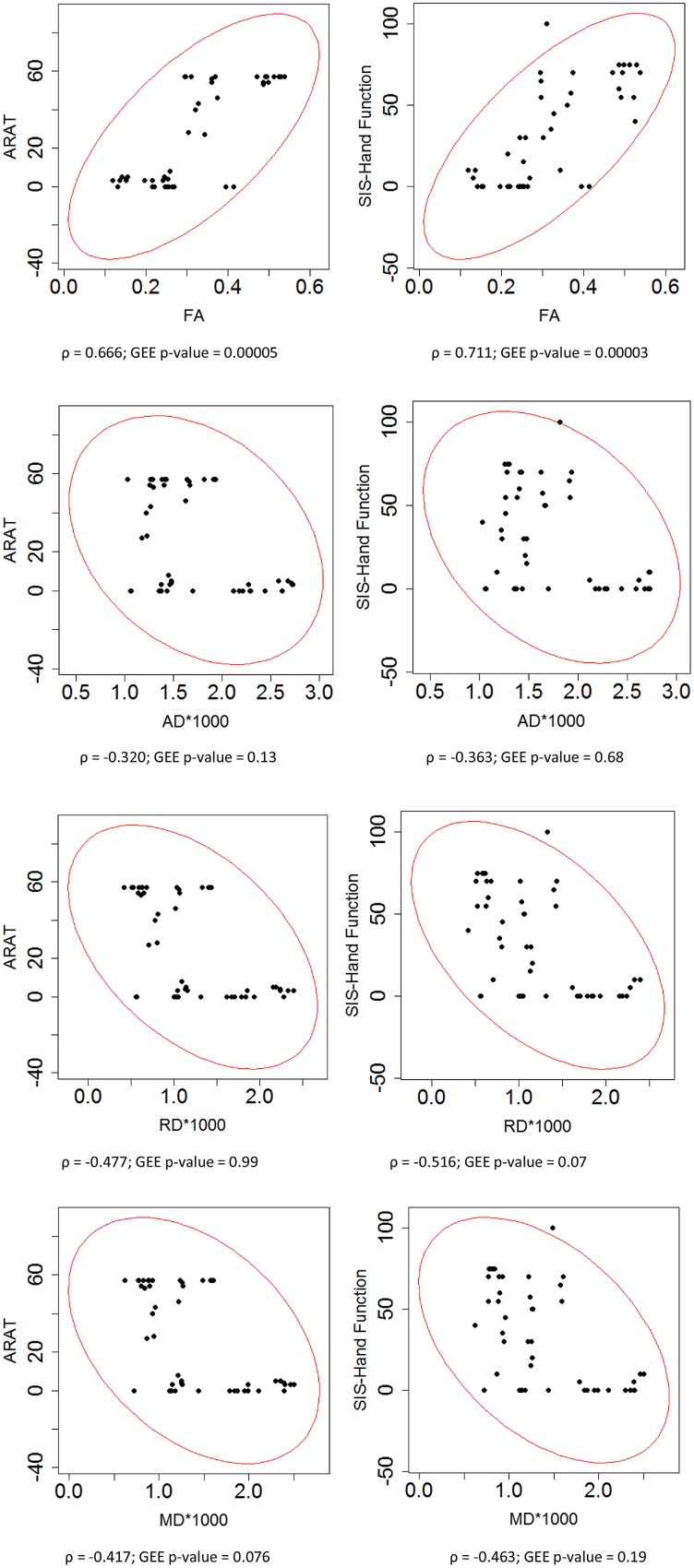
**Correlation of DTI measures and motor outcomes across time.** Each DTI measure was correlated with motor outcome measures including ARAT and SIS hand function scores assessed in impaired hand. Spearman rank correlation coefficient (ρ) and the statistical significance of correlation (GEE *p*-value) are presented.

To assess the relationship between PLIC DTI and motor outcome measures, Spearman’s rank correlation coefficients were first computed for all time points of the longitudinal data acquired from all patients. This correlation analysis showed that higher ARAT scores and higher SIS-hand function scores, indicating better performance, were significantly correlated with lower diffusivity ratios (i.e., rAD, rRD, and rMD) but higher FA ratio (i.e., rFA) in the ipsilesional PLIC (**Figure 3**). A secondary statistical analysis, the GEE analysis, was further computed to account for the dependence of repeated measurements from each patient at different time points. These analyses confirmed that the relationships observed between DTI and objective motor outcome measures (i.e., ARAT) remained statistically significant (**Table 3**). We performed the same analyses to estimate the correlations between baseline DTI measures and motor outcomes. Only baseline PLIC FA was shown to be significantly correlated with motor outcome measures (**Figure 4**).

**Table 3:** Correlation coefficients of DTI measures and motor outcomes.

Motor outcome measure	Correlation analysis	rFA	rAD	rRD	rMD
ARAT	Spearman’s rank correlation coefficients	0.603	-0.480	-0.535	-0.522
	GEE *p*-value	**0.0006**	**0.0004**	**3.15e-07**	**2.17e-06**
SIS-hand function	Spearman’s rank correlation coefficients	0.618	-0.528	-0.571	-0.569
	GEE *p*-value	**0.0002**	0.18	0.047	0.055

*Bold values indicate significant GEE tests with p-value less than 0.05.*

### Prediction of Motor Functional Recovery Using Baseline DTI Measures

**FIGURE 5:**
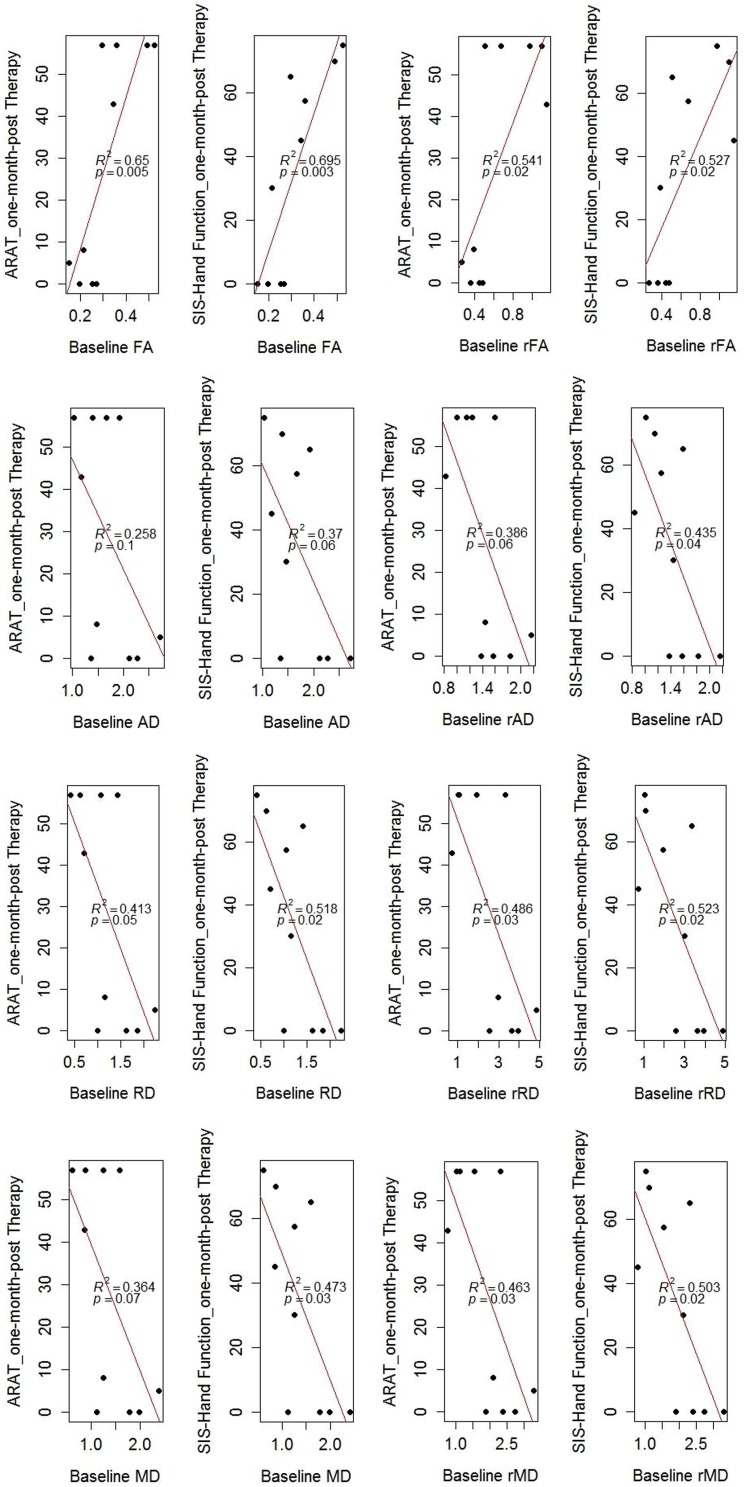
**Correlation of baseline DTI measures and motor outcomes evaluated 1-month-post intervention.** Baseline DTI measures (i.e., evaluated at immediately pre-intervention) correlated with motor outcomes measured 1-month-post intervention. *R*-squared values (*R*^2^) and *p*-values of the regression model are shown on the figures. Note: there were three patients (CI002, CI009, and CT002) missing DTI scans at 1-month-post therapy, resulting 10 data points as seens in the figures.

**FIGURE 6:**
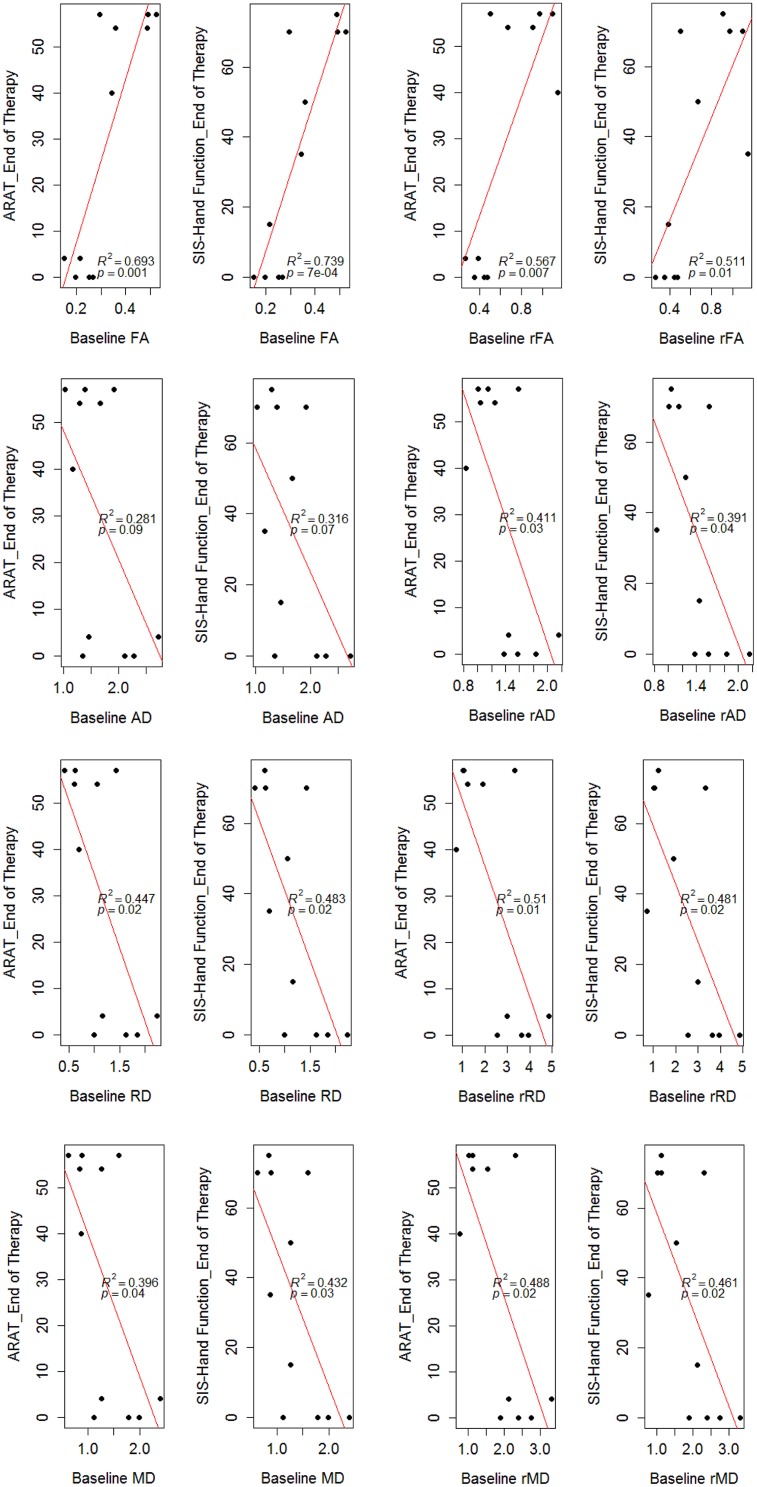
**Correlation of baseline DTI measures and motor outcomes evaluated immediately post-intervention.** Baseline DTI measures (i.e., evaluated at immediately pre-intervention) correlated with motor outcomes measured immediately post-intervention. *R*-squared values (*R*^2^) and *p*-values of the regression model are shown on the figures. There were two patients (CI002 and CI009) missing DTI scans at immediately post-therapy, resulting 11 data points as seens in the figures.

In the regression model, baseline DTI measures of the ipsilesional PLIC correlated with ARAT and SIS-hand function scores tested 1-month-post intervention (**Figure 5**). Diffusivity ratios (i.e., rAD, rRD, rMD) outperformed diffusivity measures (i.e., AD, RD, MD) in the regression model (**Figure 5**). FA remained the best predictor of motor recovery among these DTI measures (r-squared > 0.6 and *p*-value < 0.05). In addition, we examined whether baseline DTI measures of the ipsilesional PLIC were correlated with motor outcomes evaluated immediately post-intervention and observed very similar findings (**Figure 6**).

## Discussion

### PLIC in Motor Recovery

Injury to the corticospinal pathway from the primary motor cortex through the PLIC to the lower motor neurons can have significant impact on motor performance and functional recovery in stroke patients. In the majority of stroke patients, the upper-limb is more severely involved than the lower limb, with upper-extremity impairment more commonly seen with MCA strokes (Shelton and Reding, 2001). Studies using MRI suggest that motor impairment after stroke is clearly related to lesion size and location (Chen et al., 2000; Shelton and Reding, 2001), cortical activation at rest (Carter et al., 2003) and during voluntary movement (Ward et al., 2003), and the structural integrity of descending motor fibers (Pineiro et al., 2000; Shelton and Reding, 2001; Schiemanck et al., 2008). Previous studies also report that stroke location rather than the volume of an infarct is more important for predicting functional outcomes (Pineiro et al., 2000; Crafton et al., 2003). Shelton and Reding (2001) have observed that patients with purely cortical strokes have better motor outcome than patients with purely subcortical stroke. Patients with mixed cortical and subcortical stroke tended to do better than patients with purely subcortical stroke despite the expected larger size of mixed lesions (Shelton and Reding, 2001). Interestingly, they found that the PLIC was the only structure significantly associated with poor recovery of isolated upper-limb movements as assessed by the Fugl-Meyer motor score (Sanford et al., 1993). In another study investigating the predictive value of hemispheric stroke localization for the recovery of hand function 1 year post-stroke, Schiemanck et al. (2008) found that lesions of the internal capsule were associated with a significantly lower probability of return of isolated hand motor function than lesions of the cortical, subcortical regions and corona radiata. Although both observed significant relationships between lesion location (i.e., PLIC) and upper-limb motor recovery, these two studies did not quantify the structural integrity of the PLIC nor did they examine the relationship between the structural integrity of the PLIC and motor recovery. Given the significance of the PLIC to motor recovery, in this study, we systematically examined the structural integrity of the PLIC using DTI and investigated whether the structural integrity of the PLIC correlated with upper-limb motor outcomes and thereby affected motor recovery in stroke patients receiving BCI interventions. We found that the structural integrity of the PLIC was significantly affected by stroke (i.e., increased diffusivity and decreased FA values in the ipsilesional PLIC) and was significantly correlated with upper-limb motor outcomes. These observations show that upper-limb motor recovery could depend on the preservation of the PLIC, which provides important information for designing and evaluating post-stroke rehabilitation.

### Application of DTI in Stroke

Diffusion tensor imaging is a promising MRI technique for characterizing microstructural changes in many neuropathologic conditions, and has recently been applied to evaluate the relationship between the integrity of corticospinal fiber tracts and motor outcome measures in acute and chronic stroke phases (Schaechter et al., 2009; Lindenberg et al., 2010; Koyama et al., 2012). The vast majority of DTI studies on prediction of stroke outcomes have used FA. FA evaluation is often related to white matter integrity and is considered a unique and sensitive indicator of axonal loss, while its specificity for axonal loss is not clear, because it may also be sensitive to myelination status and other abnormalities (Mori, 2006). To better understand the underlying pathology in white matter integrity, several studies have evaluated directional diffusivities such as AD, RD, and MD in both animal (Song et al., 2002, 2003) and human stroke studies (Boespflug et al., 2011; Lindenberg et al., 2012; Song et al., 2012). AD has been related to axonal damage while RD is more specific to myelination. Alteration of diffusivities in the corticospinal system due to stroke has been observed in both human and animal studies (Liu et al., 2007; Kusano et al., 2009; Schaechter et al., 2009; Lindenberg et al., 2012). In our study, DTI analyses on 13 stroke patients with varying location and size of infarct in the corticospinal system yielded consistent observations – increased diffusivities and decreased FA in the ipsilesional PLIC compared with the contralesional side (**Figure 2**). This phenomenon has been suggested as a characteristic of chronic white matter degeneration (Yu et al., 2009; Lindenberg et al., 2012) and is thought to arise from the loss of the tissue’s structural integrity (Liu et al., 2007).

### Tracking Motor Recovery Using DTI

In additional to its role as a structural integrity indicator, DTI measures linearly correlate with motor impairment and motor function (Schaechter et al., 2009; Lindenberg et al., 2010, 2012; Boespflug et al., 2011; Koyama et al., 2012, 2013). Our study investigated this relationship in stroke patients receiving BCI interventions and found that ratios of DTI measures between ipsilesional and contralesional PLIC were significantly correlated with measures of motor function (i.e., ARAT) and self-reported hand impairment (i.e., SIS-hand function) during the course of intervention (**Figure 3**). More specifically, higher ARAT scores, indicating better upper-limb functioning, and higher SIS-hand function scores, indicating greater satisfaction with hand use, were significantly correlated with lower diffusivity ratio but higher FA ratio in ipsilesional PLIC. Given our observation that ipsilesional PLIC had increased diffusivity and decreased FA compared with the contralesional side, these findings suggest that the more the DTI measures of the ipsilesional PLIC resembled those observed in the contralesional PLIC, the greater the potential for functional recovery in stroke-affected limb.

We observed that ratios of diffusivity yielded stronger correlations with motor outcomes than pure diffusivity measures (**Figures 3** and **4**). This might be due to the fact that diffusivity ratios provided a normalized directional diffusivity measure by taking into account the underlying changes in contralesional side during stroke recovery. Furthermore, each diffusivity ratio correlated with motor outcomes which might indicate white matter remodeling in the ipsilesional and contralesional hemispheres during the rehabilitation period. Interestingly, both FA and rFA had a significant and strong correlation with motor outcomes. FA itself is a normalized scalar index proportional to the standard deviation of three eigenvalues, while pure diffusivity measures are combinations of eigenvalues and not normalized (Basser and Pierpaoli, 1996; Alexander et al., 2007).

### Predicting Motor Recovery Using DTI

One of the most important findings of this study is the predictive value of DTI measures to functional motor recovery following BCI facilitated FES of the impaired arm. Baseline DTI measures, especially the ratio of each DTI measure comparing the ipsilesional and contralesional PLIC, significantly predicted motor outcomes assessed 1-month-post intervention (*p*-values ≤ 0.05; **Figure 5**). Similar findings were also observed for baseline DTI measures and motor outcomes assessed immediately post-intervention (**Figure 6**). This supports the potential clinical utility of DTI measures to predict upper-limb motor functional recovery of stroke patients receiving BCI and other motor recovery interventions.

### Limitations

Small sample size (*n* = 13) and the heterogeneity of stroke patients were the primary limitations of this study. Lesion location varied in these patients. Seven of 13 patients had left hemisphere stroke and six of them had right hemisphere stroke. Within the 13 patients, three patients had subcortical stroke. A wide range of time since stroke onset was another limitation. Four patients were severely impaired and exhibited no or little improvement in functional recovery as assessed by clinical behavioral performance. These patients were minimally able to perform the designed intervention tasks, resulting in a floor effect in outcome measurements. While changes in DTI measurements were observed across time, a Kruskal–Wallis test did not show these changes to be significant. There are many factors that may result in the absence of significant changes in DTI measures across time such as the small sample size, high between-patient variance in terms of age, stroke severity and duration of stroke, and the sensitivity of the current DTI technique used in the study. Future studies will target a more homogeneous subtype of stroke patients.

There are other factors that could be useful for predicting outcomes such as age, duration of stroke, stroke severity. In the current study, we are specifically interested in the value of PLIC DTI measures in predicting motor outcomes. We performed additional analyses using age, duration of stroke (e.g., months since stroke onset) and stroke severity (e.g., baseline NIHSS-motor arm measures) as covariates, and found that ipsilesional FA, RD, and MD, age, months since stroke and motor arm scores of NIHSS baseline measures and interaction between any two of these parameters all significantly correlated with ARAT measures (*p*-values < 0.05).

The current study was initially designed as a crossover study with a small group of control subjects (see Supplementary Data). We compared motor outcome performances between the intervention (nine patients) and the control group (seven patients) who did not receive BCI intervention and found significant improvements only at mid-intervention. We also examined the relationship between FA and motor outcomes at individual time points at pre-, mid-, immediately post, and 1-month-post intervention. We observed changes in regression slope and correlation coefficients between FA and motor outcome measures across intervention and especially at immediately post intervention, suggesting that BCI intervention may have an effect on motor recovery compared to pre-intervention. These results can be seen in the Supplementary Data. It is worth noting that these results were preliminary with a small sample size and the analysis was done under the assumption of a linear relationship between FA and motor outcome measures. Additional data is needed for further validation.

## Conclusion

In this study, we investigated the relationship between DTI and motor outcome measures in order to track and predict motor functional recovery in a group of stroke patients with persistent upper extremity impairment receiving BCI intervention facilitated with FES of the impaired arm. Given the previously established significance of PLIC involvement in motor recovery, this study evaluated stroke-induced changes in structural integrity of the PLIC using DTI measures and also investigated if these changes were related to motor recovery after a course of BCI interventions. We observed that ratio of each DTI measure was significantly correlated with motor outcomes, with lower diffusivity and higher FA measures of the ipsilesional PLIC correlating with better motor recovery. Interestingly, we also observed that baseline PLIC DTI measures assessed pre-intervention significantly correlated with motor outcomes measured immediately post and 1-month-post intervention, suggesting a predictive value of PLIC DTI in motor recovery, especially in stroke patients receiving BCI-FES interventions.

## Author Contributions

JS: Assisted with patient recruitment, data collection, data analysis, and writing.

VN: Assisted with patient recruitment, data collection, and data analysis.

BY: Assisted with patient recruitment and data collection.

LW, ZN, and AR: Assisted with data collection.

MT: Provided part of system hardware and expertise.

DE: Assisted with study design and outcome measure selection and interpretation.

KC: Assisted with patient recruitment.

JAS: Assisted with study design and patient recruitment.

JW: This author is one of two lead PI’s on this project and supervised the technical and engineering aspects of the work.

VP: This author is one of two lead PI’s on this project and supervised the neuroimaging aspects of this work.

## Conflict of Interest Statement

There is one patentpending on the closed-loop neurofeedback device used for the BCI-facilitated intervention administered in this study (Pending U.S. Patent Application No. 12/715,090). This patent was filed jointly by the two lead investigators Justin C. Williams and Vivek Prabhakaran. Otherwise, the authors have no conflicts of interest to report, as this research was conducted in the absence of commercial and financial relationships that might compromise the integrity of the results reported herein.
