# Supplementary material for: DTI measures track and predict motor function outcomes in stroke rehabilitation utilizing BCI technology
Source: Front Hum Neurosci. 2015 Apr 27;9:195. doi: 10.3389/fnhum.2015.00195 (PMC4410488; doi:10.3389/fnhum.2015.00195)
Supplement: Supplementary file 1 [file Data_Sheet_1.DOCX]

**Supplementary Data:**

**DTI Measures Track and Predict Motor Function Outcomes in Stroke Rehabilitation Utilizing BCI Technology**

Jie Song^1,2^ PhD, Veena A. Nair^2^ PhD, Brittany M. Young^2,3,4^ BS, BA, Leo M. Walton^1,3^ BS, Zack Nigogosyan^2^ BS, Alexander Remsik^2^ BS, Mitchell E. Tyler^1,5^ MS, Dorothy Farrar-Edwards^6-8^ PhD, Kristin E. Caldera^5^ DO, Justin A. Sattin^8^ MD, Justin C. Williams^1,3,9^ PhD, Vivek Prabhakaran^1,2,3,4,8,10,11^ MD, PhD

^1^ Department of Biomedical Engineering, University of Wisconsin-Madison, Madison, WI, USA.

^2^ Department of Radiology, University of Wisconsin-Madison, Madison, WI, USA.

^3^ Neuroscience Training Program, University of Wisconsin-Madison, Madison, WI, USA.

^4^ Medical Scientist Training Program, University of Wisconsin-Madison, Madison, WI, USA.

^5^ Department of Orthopedics and Rehabilitation, University of Wisconsin-Madison, Madison, WI, USA.

^6^ Departments of Kinesiology, University of Wisconsin-Madison, Madison, WI, USA.

^7^ Departments of Medicine, University of Wisconsin-Madison, Madison, WI, USA.

^8^ Department of Neurology, University of Wisconsin-Madison, Madison, WI, USA.

^9^ Department of Neurosurgery, University of Wisconsin-Madison, Madison, WI, USA.

^10^ Department of Psychiatry, University of Wisconsin-Madison, Madison, WI, USA.

^11^ Department of Psychology, University of Wisconsin-Madison, Madison, WI, USA.

**Changes in motor outcomes**

Our current study is limited by a relatively small sample size (n = 13) and the heterogeneity of these stroke patients in terms of age, stroke severity and duration of stroke. We performed Kruskal–Wallis tests on DTI and motor outcome measures and did not find these measures change significantly across intervention time (i.e., immediately pre-, mid-, immediately post- and one-month-post BCI intervention).

Despite that, this study was initially designed as a crossover study which means there were patients who assigned to control group would not receive BCI intervention from the very beginning of their participation (Song et al., 2014). These patients will be crossed-over to receive intervention once the control period is over. However, as the intervention period takes approximately a month, thus data collection is taking a relatively long period. By the time this manuscript was written, we only have 7 control patients from the control group who completed the study (3 of 7 don’t have DTI imaging data). There were 9 patients from the intervention group who received intervention immediately after joining the study and completed the study (Table S1). We examined changes of motor outcomes compared to baseline (i.e., immediately pre-intervention) at mid-, immediately post- and one-month-post BCI intervention between the intervention and control groups. We found that at mid-intervention, there was significant increase in measures of SIS-hand function and ARAT (2-sample t-test, one-tailed, p-value = 0.016 for SIS-hand function and 0.032 for ARAT) in the intervention group. This appears to suggest that BCI intervention is having an effect on motor recovery reflected by motor outcome changes compared to baseline.

**Correlation of FA and motor outcomes at individual time points**

As this study was designed with longitudinal and repeated measurements, we used a generalized estimating equation (GEE) for regression analysis which takes into account the dependency of repeated measurements from the same patient in the regression analysis and thus allow for evaluation of the relationship between DTI and motor outcome measures longitudinally. In addition, we conducted correlation and linear regression analysis on FA and motor outcome measures at individual time points from those 13 patients with DTI imaging data (Table S1). We observed an increase in correlation coefficients from pre-intervention to immediately post-intervention and a decrease at one-month-post intervention compared to pre-intervention (Table S2). We also observed that the regression slope of FA and SIS-hand function increased at mid-, immediately post- and one-month-post intervention compared to baseline. However, there was no major change in the regression slope of FA and ARAT (Table S3). There may be a difference in the FA value and its relationship to subjective (SIS-hand function) vs. objective (ARAT) motor assessments. Therefore we further compared the r-squared values which indicates to what extent that the FA linear model explains the variation of the motor outcome measures (Table S3). We found that more of the motor performances (SIS-hand function and ARAT) is accounted for by FA in terms of variance at immediately post-intervention in comparison to pre-intervention. At one-month-post intervention, there was less accountability by FA in terms of variance than there was at pre-intervention. There may be practice effects on these tasks that may be playing a role in accounting for the behavioral performances.

Table S1: Clinical motor outcome assessments of the stroke-affected limb from experimental and control groups.

| **Groups** | **Subject ID** | **Stroke affected limb** | **Time points** | **ARAT** | **SIS-**  **Hand Function** |
| --- | --- | --- | --- | --- | --- |
| **Intervention Group** | CI001 | R | Pre-therapy | 0 | 0 |
|  |  |  | Mid-therapy | 3 | 0 |
|  |  |  | Immediately post-therapy | 0 | 0 |
|  |  |  | one-month-post-therapy | 0 | 0 |
|  | CI002 | R | Pre-therapy | 0 | 0 |
|  |  |  | Mid-therapy | 0 | 0 |
|  |  |  | Immediately post-therapy | 0 | 0 |
|  |  |  | one-month-post-therapy | 0 | 0 |
|  | CI003 | R | Pre-therapy | 57 | 40 |
|  |  |  | Mid-therapy | 57 | 55 |
|  |  |  | Immediately post-therapy | 57 | 70 |
|  |  |  | one-month-post-therapy | 57 | 75 |
|  | CI004 | R | Pre-therapy | 3 | 0 |
|  |  |  | Mid-therapy | 0 | 0 |
|  |  |  | Immediately post-therapy | 0 | 0 |
|  |  |  | one-month-post-therapy | 0 | 0 |
|  | CI005 | R | Pre-therapy | 56 | 50 |
|  |  |  | Mid-therapy | 46 | 70 |
|  |  |  | Immediately post-therapy | 54 | 50 |
|  |  |  | one-month-post-therapy | 57 | 57.5 |
|  | CI007 | R | Pre-therapy | 57 | 55 |
|  |  |  | Mid-therapy | 57 | 100 |
|  |  |  | Immediately post-therapy | 57 | 70 |
|  |  |  | one-month-post-therapy | 57 | 65 |
|  | CI008 | L | Pre-therapy | 3 | 0 |
|  |  |  | Mid-therapy | 5 | 0 |
|  |  |  | Immediately post-therapy | 4 | 0 |
|  |  |  | one-month-post-therapy | 5 | 0 |
|  | CI009 | L | Pre-therapy | 3 | 0 |
|  |  |  | Mid-therapy | 3 | 10 |
|  |  |  | Immediately post-therapy | 0 | 5 |
|  |  |  | one-month-post-therapy | 3 | 10 |
|  | CI010 | L | Pre-therapy | 57 | 55 |
|  |  |  | Mid-therapy | 54 | 60 |
|  |  |  | Immediately post-therapy | 57 | 70 |
|  |  |  | one-month-post-therapy | 57 | 70 |
| **Control group** | CT001 | L | Control point 1 | 0 | 0 |
|  |  |  | Control point 2 | 0 | 0 |
|  |  |  | Control point 3 | 0 | 0 |
|  |  |  | Control point 4 | 0 | 5 |
|  | CT002 | R | Control point 1 | 54 | 55 |
|  |  |  | Control point 2 | 57 | 35 |
|  |  |  | Control point 3 | 51 | 50 |
|  |  |  | Control point 4 | 53 | 75 |
|  | CT003 | L | Control point 1 | 26 | 10 |
|  |  |  | Control point 2 | 27 | 0 |
|  |  |  | Control point 3 | 32 | 20 |
|  |  |  | Control point 4 | 27 | 10 |
|  | CT004 | R | Control point 1 | 54 | 30 |
|  |  |  | Control point 2 | 57 | 45 |
|  |  |  | Control point 3 | 57 | 45 |
|  |  |  | Control point 4 | 54 | 55 |
|  | CT006 | R | Control point 1 | 0 | 15 |
|  |  |  | Control point 2 | 0 | 10 |
|  |  |  | Control point 4 | 0 | 0 |
|  | CT008 | L | Control point 1 | 0 | 0 |
|  |  |  | Control point 2 | 0 | 0 |
|  |  |  | Control point 3 | 0 | 0 |
|  |  |  | Control point 4 | 3 | 0 |
|  | CT010 | L | Control point 1 | 2 | 25 |
|  |  |  | Control point 2 | 9 | 0 |
|  |  |  | Control point 3 | 3 | 35 |
|  |  |  | Control point 4 | 3 | 20 |

Table S2: Correlation analysis of FA and motor outcome measures.

| Motor Outcomes | Time points | Spearman’s rank correlation coefficient | p-values |
| --- | --- | --- | --- |
| ARAT | Pre-therapy | 0.75 | 0.008 |
|  | Mid-therapy | 0.62 | 0.023 |
|  | Immediately post-therapy | 0.80 | 0.002 |
|  | one-month-post-therapy | 0.55 | 0.063 |
| SIS-Hand function | Pre-therapy | 0.76 | 0.006 |
|  | Mid-therapy | 0.61 | 0.026 |
|  | Immediately post-therapy | 0.83 | 0.001 |
|  | one-month-post-therapy | 0.65 | 0.023 |

Table S3: Linear regression analysis of FA and motor outcome measures.

| Motor Outcomes | Time points | Regression slope | p-values | R-squared |
| --- | --- | --- | --- | --- |
| ARAT | Pre-therapy | 175.48 | 0.002 | 0.65 |
|  | Mid-therapy | 154.20 | 0.002 | 0.56 |
|  | Immediately post-therapy | 169.93 | 0.001 | 0.66 |
|  | one-month-post-therapy | 144.31 | 0.021 | 0.37 |
| SIS-Hand function | Pre-therapy | 163.94 | 0.006 | 0.54 |
|  | Mid-therapy | 168.35 | 0.023 | 0.33 |
|  | Immediately post-therapy | 208.78 | 0.0003 | 0.72 |
|  | one-month-post-therapy | 171.66 | 0.016 | 0.40 |
